# Minimum values for midluteal plasma progesterone and estradiol concentrations in patients who achieved pregnancy with timed intercourse or intrauterine insemination without a human menopausal gonadotropin

**DOI:** 10.1186/s13104-018-3188-x

**Published:** 2018-01-22

**Authors:** Yukiko Takaya, Hidehiko Matsubayashi, Kotaro Kitaya, Rie Nishiyama, Kohei Yamaguchi, Takumi Takeuchi, Tomomoto Ishikawa

**Affiliations:** Reproduction Clinic Osaka, 15F, Grand Front Osaka Tower A4-20 Ofukacho, Kita, Osaka, 530-0011 Japan

**Keywords:** Estradiol, Implantation, Natural cycle, Pregnancy, Progesterone

## Abstract

**Objective:**

The aim of the study was to assess the lower limits of midluteal plasma progesterone and estradiol concentrations in patients who achieved pregnancy with timed intercourse or intrauterine insemination without a human menopausal gonadotropin stimulation.

**Results:**

We included 297 pregnant cycles of 297 women and assessed midluteal plasma progesterone and estradiol concentrations and pregnancy outcomes, retrospectively. These cycles were compared with the non-pregnant cycles (406 cycles) of the same women who became pregnant. Mean midluteal plasma P4 and E2 concentrations were significantly (P < 0.01) higher in pregnant cycles (14.5 and 188.5 pg/mL) than in non-pregnant cycles (10.7 and 162.6 pg/mL). The 5 percentiles of progesterone and estradiol in pregnant cycles were 5.6 and 70.2 pg/mL, respectively. The lowest progesterone and estradiol levels in pregnant cycles were 2.3 and 23.4 pg/mL, respectively. In non-pregnant cycles, many women had low P4 levels that were less than 5.6 ng/mL. Subgroup analyses showed slight differences among the four groups, which may have been due to the ovarian function of each group. Miscarriage was not related to progesterone and estradiol concentrations. These values may be useful for the evaluation of necessary values for pregnancy with timed intercourse or intrauterine insemination.

## Introduction

Progesterone (P4) and estradiol (E2) are important for a normal menstrual cycle, implantation, and early embryonic development [1]. Since inadequate levels of these hormones (particularly P4) may lead to unsuccessful pregnancy and/or miscarriage [1], luteal phase deficiency (LPD) has been described and discussed. However, a diagnostic test for LPD has not yet been proven to be reliable in clinical settings (e.g., basal body temperature charting, urinary luteinizing hormone detection kits, luteal P4 levels, and endometrial biopsy) [1].

The midluteal plasma P4 concentration is considered to be one of the best markers for ovulation and luteinization because P4 may reflect the functions of the corpus luteum [2–4]. However, the optimal times to conduct measurements and the methods by which to evaluate P4 values have not yet been established, and the minimum P4 concentration that defines fertile luteal function currently remains unknown. To the best of our knowledge, only one previous study reported reference values for midluteal P4 in 192 patients who achieved pregnancy with human menopausal gonadotropin (hMG)-stimulated cycles [5]. Since that study included 48 multiple pregnancies and hMG (150 IU) was administered daily, the mean value for P4 was relatively high at 29.07 ng/mL. The minimum value for full-term singleton pregnancy (N = 72) was concluded to be 10.83 ng/mL [5]. Besides P4, E2 is regarded as a marker for ovulation and luteinization [6]. We conducted this study in order to identify minimum values for P4 and E2 in patients who became pregnant with timed intercourse (TI) or intrauterine insemination (IUI) without hMG stimulation, because a hMG stimulation leads to increases in P4 and E2 [1]. If it is possible to establish these values, they might be useful for evaluations of the necessary values for pregnancy with not only TI or IUI, but also natural cycle-frozen embryo transfer (FET) with the same protocol.

## Main text

### Materials and methods

A single-center retrospective study was performed. All patients gave written informed consent for this study. Institutional Review Board (IRB) approval was obtained from Jinjukai Ishikawa Hospital (the mother organization of Reproduction Clinic Osaka).

After opening of our institute on October 2013, we have been analyzing midluteal plasma P4 and E2 for all women who hoped for children in all cycles of TI or IUI. At the time of venipuncture, we asked patients whether they wanted to participate in the present study, and those who provided written informed consent were included. Since the purpose of this study is to determine the lower limit of P4 and E2 in order to achieve pregnancy, we excluded the cycles without pregnant. Between October 2013 and December 2016, 307 women (all Japanese) who became pregnant with TI or IUI were included in the present study. After exclusion of 4 twin pregnancies and 6 ectopic pregnancies, 297 cycles were evaluated for analyses. As a comparison group, we used the non-pregnant cycles of the same women who became pregnant. We included a natural cycle or minimum ovarian stimulation for follicle growth. Ovulation induction cycles with hMG were excluded. The minimum ovarian stimulation included letrozole (2.5 mg × 2–4 days, Novartis Pharma K.K., Tokyo, Japan), cyclofenil (300–600 mg × 5 days, Aska Pharmaceutical Co., Ltd., Tokyo, Japan), and clomifene (50 mg × 3–5 days, Fuji Pharma Co., Ltd., Tokyo, Japan). Follicular development was monitored by vaginal ultrasonography (LOGIQ A5, GE Healthcare Japan, Tokyo, Japan). Cycles with three or more developing follicles were canceled. In all cycles, gonadotropin releasing hormone agonist (GnRHa, buserelin acetate 600 μg, Fuji Pharma Co., Ltd) was administered as a trigger. Ovulation was confirmed by ultrasound and serum samples were collected on the 7th day after ovulation. Blood was collected from the forearm of patients with a 21G needle. After venipuncture, blood was centrifuged at 3800 rpm × 5 min (Mode 2420, Kubota Corporation, Gunma, Japan), and serum samples were shipped to a clinical laboratory company (Medic Co., Shiga, Japan) on the same day. Serum P4 and E2 concentrations were measured by the company (Medic Co.) using a chemiluminescent enzyme immunoassay (progesterone and estradiol II, Architect^®^, Abott Japan Co., Ltd., Tokyo, Japan). Cycles with luteal support were excluded. We retrospectively evaluated the minimum levels of E2 and P4 in the cycle achieving pregnancy. Miscarriage was defined as a missing or undetectable heart beat at 12 weeks of gestation, while ongoing pregnacy was defined as a confirmed heart beat beyond 12 weeks of gestation.

The Kruskal–Wallis H-test (two-sided) was used for comparisons among three or more groups. The Mann–Whitney U-test (two-sided) was used for comparisons between two groups. Correlations were detected by Pearson’s test. Significance was defined as P < 0.05.

### Results

In this cohort, 297 pregnant cycles of 297 women were evaluated for analyses, and were compared with the non-pregnant cycles (406 cycles) of the same women who became pregnant. Patient profiles and endocrine parameters were shown in Table 1. No significant differences were observed in age or BMI between the two groups. Mean midluteal plasma P4 and E2 concentrations were significantly (P < 0.01) higher in pregnant cycles (14.5 and 188.5 pg/mL) than in non-pregnant cycles (10.7 and 162.6 pg/mL). The 5 percentiles of P4 and E2 in pregnant cycles were 5.6 and 70.2 pg/mL, respectively. The lowest P4 and E2 levels in pregnant cycles were 2.3 and 23.4 pg/mL, respectively.

**Table 1:** Patient profiles and endocrine parameters

	Pregnant	Non-pregnant	P value
Patient number	297	159	
Cycle number	297	406	
Age (years)*	33.4 ± 4.0	33.6 ± 3.8	NS
BMI (kg/m^2^)*	20.7 ± 2.8	20.7 ± 2.7	NS
P4 (ng/mL)			
Mean*	14.5 ± 7.6	10.7 ± 8.0	< 0.01
Median	12.6	9.7	NA
5‰ P4	5.6	0.54	NA
Lowest P4	2.3	0.1	NA
E2 (pg/mL)			
Mean*	188.5 ± 118.8	162.6 ± 107.8	< 0.01
Median	152.1	134.1	NA
5‰ E2	70.2	42.8	NA
Lowest E2	23.4	25.3	NA

*NS* not significant; *NA* = not available

* Values are indicated by means ± standard deviations. The Mann–Whitney U-test (two-sided) was used for comparisons between the two groups

Pregnant cycles were divided into four groups (letrozole 26 cycles, cyclofenil 75 cycles, clomifene 132 cycles, no medication 64 cycles) for the subgroup analyses (Table 2). No significant difference was observed in BMI among the four groups. The letrozole group (31.2 years) was significantly younger than the clomifene group (33.9 years, P < 0.01) and the cyclofenil group (33.8 years, P < 0.05). Mean midluteal plasma P4 concentrations were significantly higher in the clomifene group (17.2 ng/mL) than in the cyclofenil group (12.0 ng/mL, P < 0.01) and no medication group (12.8 ng/mL, P < 0.05). Mean midluteal plasma E2 concentrations were significantly higher (P < 0.01) in the clomifene group (241.8 pg/mL) than in the letrozole (127.7 pg/mL), cyclofenil (146.9 pg/mL), and no medication (149.5 pg/mL) groups.

**Table 2:** Subgroup analyses in pregnant cycles

	Letrozole	Cyclofenil	Clomifene	No medication	P value
	(n = 26)	(n = 75)	(n = 132)	(n = 64)	
Age (years)*	31.2 ± 3.0^ab^	33.8 ± 3.9	33.9 ± 4.0	33.4 ± 4.2	< 0.01
BMI (kg/m^2^)*	20.5 ± 3.6	20.4 ± 2.4	21.1 ± 2.8	20.4 ± 2.9	NS
P4 (ng/mL)					
Mean*	12.8 ± 4.1	12.0 ± 5.9^a^	17.2 ± 9.0	12.8 ± 5.4^c^	< 0.01
Median	12.9	11.1	15.4	11.9	NA
5‰ P4	5.9	4.8	6.6	5.8	NA
Lowest P4	4.6	2.3	3.7	5.0	NA
E2 (pg/mL)					
Mean*	127.7 ± 84.6^a^	146.9 ± 80.6^a^	241.8 ± 138.7	149.5 ± 56.9^a^	< 0.01
Median	106.8	133.1	203.3	140.1	NA
5‰	28.2	59.9	93.3	71.3	NA
Lowest E2	23.4	44.2	70.2	71.1	NA

*NS* not significant, *NA* not available

* Values are indicated by means ± standard deviations. The Kruskal–Wallis H-test (two-sided) was used for comparisons among the four groups

^a^The Mann–Whitney U-test (two-sided) was used for comparisons between the two groups compared with clomifene group (P < 0.01)

^b^The Mann–Whitney U-test (two-sided) was used for comparisons between the two groups compared with cyclofenil group (P < 0.05)

^c^The Mann–Whitney U-test (two-sided) was used for comparisons between the two groups compared with clomifene group (P < 0.05)

**Fig. 1:**
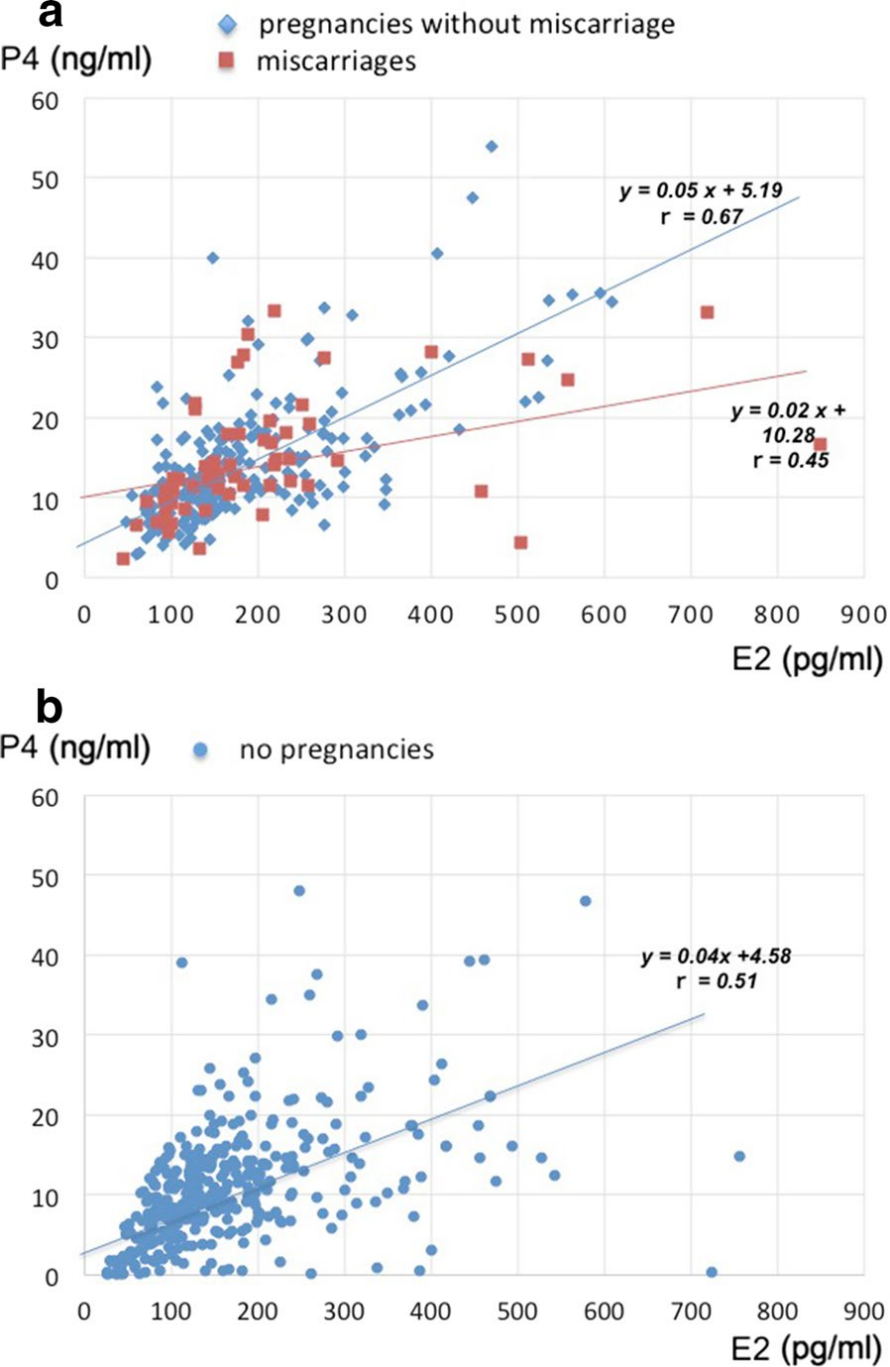
**a** Scatter plot for E2 and P4 concentrations in pregnant cycles without letrozole. Red squares indicate miscarriages (missing or undetectable heart beat at 12 weeks of gestation), and blue diamonds indicate ongoing pregnancies as a confirmed heart beat beyond 12 weeks of gestation. **b** Scatter plot for E2 and P4 concentrations in non-pregnant cycles without letrozole

Since a previous study reported that letrozole decreases E2 levels [7], we evaluated the data with or without letrozole in pregnant cycles. A correlation between E2 and P4 was observed in cycles without letrozole (R = 0.60, P < 0.01), but not in cycles with letrozole (R = 0.13). According to the scatter plot for E2 and P4 in pregnant cycles without letrozole (Fig. 1a), miscarriage (red squares) was not associated with either E2 or P4 concentrations. However, P4 in miscarriage group was slightly reduced compared with ongoing pregnancy group (blue diamonds) without statistical significance. As a control group, Fig. 1b shows a scatter plot for E2 and P4 in non-pregnant cycles without letrozole. Many women had low P4 levels less than 5.6 ng/mL. However, some women also had high P4 levels.

### Discussion

Our results suggest that the lower limits (5‰) of midluteal plasma P4 and E2 in patients who became pregnant with TI or IUI without hMG stimulation were 5.6 and 70.2 pg/mL, respectively. This is the first study to report reference values for P4 and E2 in patients with pregnancy without an hMG stimulation. Only one previous study showed the minimum value of midluteal P4 in 72 patients who achieved a singleton pregnancy in stimulated cycles (hMG 150 IU daily) with timed intercourse as 10.8 ng/mL [5]. We set the lower limit of P4 and E2 as 5‰, because the World Health Organization (WHO) determined semen quality parameters as 5‰ for the lower reference limit [8].

As minimum values, ovulation and luteinization are confirmed when midluteal P4 was more than 1.8–5.0 ng/mL [9–13]; however, it has yet to be established whether these values are adequate for pregnancy. Since pregnancy is the best evidence for adequate ovulation and luteinization, reference values for P4 and E2 need to be evaluated in pregnant cycles only. In non-pregnant cycles, many women had low P4 levels less than 5.6 ng/mL, while some had high P4 levels; other factors may have contributed to the failure to achieve pregnancy in the latter.

Recent evidence was obtained to show reference values for P4. The nucleolar channel system (NCS) in the endometrium is a marker for endometrial receptivity [14]. An NCS study in the endometrium revealed that the minimum P4 level was 4.0 ng/mL [15]. This value indicated ovulation only; it did not indicate the necessary value for pregnancy. Since there is no linear correlation between the prevalence of NCS and P4 levels, this level simply indicated the P4 threshold level. Thus, endometrial receptivity may require 4.0 ng/mL or more of P4.

In terms of a luteal phase deficiency, there is currently no standard P4 value during the luteal phase in normal fertile women [1, 16, 17]. Therefore, whether the minimum P4 concentration defines fertile luteal function remains unknown. Moreover, corpus luteum functions vary from cycle to cycle because the corpus luteum changes from cycle to cycle [2, 3]. Since it currently remains unclear whether women have a luteal phase deficiency [1], a minimum value may be useful as a treatment tool.

Subgroup analyses showed slight differences among the four groups, which may have been due to the ovarian function of each group differing at the time of the selection of medication by the physician. However, the number of samples in each group was too small to reach a concrete conclusion. Therefore, we included all women in the present study.

### Conclusions

The lower limits of midluteal plasma P4 and E2 concentrations in patients who achieved pregnancy with TI or IUI without an hMG stimulation were 5.6 ng/mL and 70.2 pg/mL, respectively, in our limited study population.

### Limitations

This was a retrospective observational study. A prospective study may be difficult to perform to assess minimum values for pregnancy because data from pregnant patients only are needed for analyses. Furthermore, since P4 levels may fluctuate in the 90-min period during the midluteal phase (2.3–40.1 ng/mL) [18], a single value may not be sufficient. However, since the aim of the present study was to assess the minimum values of P4 and E2, fluctuations may not have influenced the data obtained. Moreover, the sample size may have been too small to establish the minimum values of P4 and E2, and this was a single-center study conducted in the Osaka region using a single ethnicity (only Japanese). Therefore, further studies are needed in order to obtain minimum values in ethnic groups other than Japanese.

## Abbreviations

E2: estradiol
FET: frozen embryo transfer
hMG: human menopausal gonadotropin
IUI: intra uterine insemination
LPD: luteal phase deficiency
NCS: nucleolar channel system
P4: progesterone
TI: timed intercourse
